# Evaluating plantar correction strategies in pediatric growing pains: a postural and pain analysis in 647 children

**DOI:** 10.25122/jml-2025-0084

**Published:** 2025-05

**Authors:** Cezar Ionita, Stefan Tiron, Summer Abdallah, Silvia-Andreea Gheorghe-Simionesie, Dorin Simionesie, Brindusa Ilinca Mitoiu, Oana Belei, Ileana Enatescu, Felix Bratosin, Adriana Sarah Nica

**Affiliations:** 1 Department of Occlusion and Fixed Prosthodontics, Faculty of Dental Medicine, Carol Davila University of Medicine and Pharmacy, Bucharest, Romania; 2 Dr. Tiron Medical Center, Bucharest, Romania; 3 University of St. Augustine for Health Sciences, Coral Gables, FL, USA; 4 Advanced Semiconductor Materials Lithography “ASML”, Veldhoven, Netherlands; 5 Department of Rehabilitation, Carol Davila University of Medicine and Pharmacy, Bucharest, Romania; 6 First Pediatric Clinic, Disturbances of Growth and Development on Children Research Center, Victor Babeș University of Medicine and Pharmacy, Timișoara, Romania; 7 Discipline of Neonatology, Victor Babeș University of Medicine and Pharmacy, Timișoara, Romania; 8 Methodological Research Center for Infectious Diseases, Victor Babeș University of Medicine and Pharmacy, Timișoara, Romania

**Keywords:** growing pains, posture, foot pressure distribution, insoles, children, stabilometry

## Abstract

Growing pains affect many children, but their underlying mechanisms are not fully understood. Potential contributors include anatomical malalignment and abnormal foot-pressure distribution (FPD). This study evaluated: (1) whether age, sex, and handedness correlate with growing-pain intensity, (2) whether anterior or posterior foot loading predominates in these children, and (3) whether customized plantar insoles improve subjective pain and objective postural or gait parameters. A total of 647 children (ages 3–14 years) underwent baseline stabilometric testing (Freemed platform) and were classified as anterior or posterior loaders. Pain status was recorded (absent, mild, moderate, intense). Custom insoles were prescribed for significant anomalies; 178 children returned for follow-up, including 137 who repeated platform assessments, and 178 who were reassessed for pain. Additional subgroup analyses examined insole compliance (high vs. low) and gait changes (step length, stance width, foot progression angle, single-limb stance, gait velocity). Of the initial 647 children, 87% demonstrated posterior orientation, and 55% reported some level of pain. No significant correlations emerged between pain intensity and age, sex, or handedness. Among the 178 reassessed patients, those with higher insole compliance and posterior orientation showed the greatest pain relief (up to 81.8% improvement), while lower-compliance subgroups reported 54.2–62.5% improvement (*P* = 0.021). Objective FPD improved in 67.7% of the 'Posterior + High' group versus 46.7% of the 'Anterior + Low' group (*P* = 0.043). Gait analysis revealed significant improvements in step length, stance width, single-limb stance, and gait velocity (*P* < 0.05) among children with baseline pain. Posterior foot loading was prevalent in this cohort, and personalized insole therapy was associated with meaningful improvements in subjective pain reports and quantitative gait parameters. While causality cannot be confirmed by this observational design, the findings suggest that targeted plantar corrections may mitigate growing pains and enhance postural stability.

## INTRODUCTION

Growing pains, described in children ages 3–14, remain a vexing clinical phenomenon [1,2]. Although often attributed to normal growth processes, many mechanistic theories have been proposed, including muscle fatigue, genetic predisposition, anatomical malalignment, or even psychological factors [3]. These pains are typically localized to the lower limbs and may disturb sleep or daily activities.

One area of interest is the role of foot posture and plantar pressures in mediating musculoskeletal stresses. Research indicates that foot pressure distribution (FPD) and center-of-pressure displacements can reveal underlying postural or neuromuscular dysfunction [1,2]. Excessive plantar loading may contribute to conditions like pes planus, valgus knees, scoliosis, and hyperkyphosis [4-9], as well as be linked to pain in multiple anatomical sites [10-13].

In recent years, computerized baropodometric analysis has facilitated the study of static and dynamic posture in children. By assessing FPD, clinicians can prescribe customized orthoses or insoles to correct foot orientation, particularly in the anteroposterior dimension, to mitigate growing pains [14-17]. However, evidence clarifying the mechanisms of action and the magnitude of symptom relief remains limited [18-25].

Accordingly, our overarching hypothesis proposes that anteroposterior postural correction through personalized plantar insoles can induce clinically meaningful improvements in subjective pain levels and stabilometric parameters among children with growing pains. We sought to (1) characterize demographic and clinical factors associated with growing pains, (2) examine foot-pressure orientation (anterior vs. posterior) in children with and without pain, and (3) evaluate the efficacy of insole-based interventions in improving FPD and alleviating pain.

## MATERIAL AND METHODS

### Study design and participants

We conducted a retrospective observational study at a specialized paediatric orthopaedics clinic (Dr. Tiron Medical Center, Bucharest, Romania). Over four years (2018–2022), 647 children aged 3–14 years were evaluated. Inclusion criteria were: (1) age between 3 and 14 years, (2) stable health history without major neurological or vestibular pathologies, and (3) availability of stabilometric measurements and documented pain status. Exclusion criteria included incomplete records or confirmed systemic conditions affecting balance (e.g., central vertigo, known vestibular disease).

Children were subdivided into three widely accepted developmental groups: 3–6, 7–10, and 11–14 years, to align with recognized phases of growth and pubertal transition [18,26,27]. The consulting physician classified pain intensity (absent, mild, moderate, or intense) based on parental reports and/or the child’s self-report [28,29]. Demographic data (age, sex, dominant hand) were noted.

### Stabilometric analysis and plantar corrective intervention

All participants underwent baseline baropodometric assessment on a Freemed platform [24]. The system measured static foot pressure distribution in a standardized stance (barefoot, eyes open, arms at sides). Foot orientation was defined as anterior if the algebraic sum of forefoot pressures exceeded 50% of total foot load, or posterior if hindfoot pressures dominated.

Children with significant static or dynamic foot anomalies, as assessed by the physician, were offered individualized insoles. These insoles were fabricated using software-integrated algorithms that processed plantar load data to create customized arch support and corrective elements. Subjects were instructed to wear insoles daily, except during sleep or bathing.

Follow-up intervals ranged from 2 to 6 months, depending on clinical indications. At reassessment, stabilometric testing was repeated in 129 participants with a posterior orientation at baseline. Pain status was again categorized in the same absent/mild/moderate/intense scheme. A similar evaluation was carried out in 59 children who had no pain at baseline (control subgroup).

### Data collection and variables

In the study, demographic data, including age (measured in years), sex, and dominant hand (categorized as right, left, or ambidextrous), were collected. The foot pressure distribution (FPD) was analyzed to determine the anterior versus posterior orientation, based on whether hindfoot pressure exceeded 50% or was less than 50% of the total foot load. Pain intensity was categorized into four levels: absent (ABS), mild (MI), moderate (MO), or intense (INT).

### Statistical analysis

Subgroup comparisons were made to evaluate the presence versus absence of pain and changes in FPD measurements over time, specifically noting improvements, stationarity, or aggravation. The correlation and risk analyses involved calculating Pearson’s or Spearman’s correlation coefficients to assess the relationships between pain intensity and variables such as age, sex, dominant hand, and orientation. Additionally, a logistic regression model was employed to test the contribution of key variables, including sex, age group, orientation, and handedness, with the presence or absence of pain as the outcome variable.

Data were summarized with means, standard deviations, and percentages. Group comparisons (pain vs. no pain, age categories, etc.) used chi-square or Fisher’s exact tests for categorical variables. Correlations (r) were interpreted using standard thresholds (|r| < 0.5, signifying weak association). Logistic regression generated odds ratios (OR) with 95% confidence intervals (CI). A *P* value <0.05 was deemed significant. SPSS (version 27) was used for all analyses.

## RESULTS

The study population comprised 647 children with a mean age of 8.14 ± 2.43 years, distributed across three age groups: 3–6 years (202 children, 31.2%), 7–10 years (270 children, 41.7%), and 11–14 years (175 children, 27.1%). The sample showed a slight male predominance with 342 boys (52.9%) compared to 305 girls (47.1%). In terms of handedness, the vast majority of the children were right-handed (561, 86.7%), with left-handed (62, 9.6%) and ambidextrous (24, 3.7%) children representing smaller proportions. Regarding pain intensity, 289 children (44.7%) reported no pain, while the rest experienced varying degrees of pain: mild pain was reported by 104 children (16.1%), moderate by 188 (29.1%), and intense by 66 (10.2%). Orientation analysis showed that most children had a posterior orientation (562 children, 86.9%), with a smaller number showing an anterior orientation (85 children, 13.1%), as presented in Table 1.

**Table 1:** Baseline demographics of the study population (n = 647)

Characteristic	*n* (%)
**Age (mean ± SD)**	8.14 ± 2.43 years
**Age group**	
3–6 years	202 (31.2)
7–10 years	270 (41.7)
11–14 years	175 (27.1)
**Sex**	
Male	342 (52.9)
Female	305 (47.1)
**Dominant hand**	
Right (RH)	561 (86.7)
Left (LH)	62 (9.6)
Ambidextrous (AMB)	24 (3.7)
**Pain intensity**	
Absent (ABS)	289 (44.7)
Mild (MI)	104 (16.1)
Moderate (MO)	188 (29.1)
Intense (INT)	66 (10.2)
**Orientation**	
Posterior (> 50% rearfoot)	562 (86.9)
Anterior (≤ 50% rearfoot)	85 (13.1)

Age distribution was similar between the groups, with children aged 3-6 years making up 30.7% and 31.8%, respectively, 7-10 years at 42.2% and 41.2%, and 11-14 years at 27.1% and 27.0%. Sex distribution also showed no significant difference, with males comprising 51.4% in the pain group and 54.7% in the no-pain group (*P* = 0.541). The hand dominance variable exhibited no significant difference (*P* = 0.8), with right-handed children representing 86.6% and 86.9% of the pain and no-pain groups, respectively. Orientation showed a minor variation, where 85.5% of the pain group had a posterior orientation compared to 88.6% in the no-pain group; however, this was not statistically significant (*P* = 0.112), as described in Table 2.

**Table 2:** Subgroup comparison: presence vs. absence of baseline pain

Variable	Pain (*n* = 358)	No pain (*n* = 289)	*P* value (χ^2^/Fisher)
**Age group**			
3–6 years	110 (30.7)	92 (31.8)	0.728
7–10 years	151 (42.2)	119 (41.2)	
11–14 years	97 (27.1)	78 (27.0)	
**Sex**			0.541
Male	184 (51.4)	158 (54.7)	
Female	174 (48.6)	131 (45.3)	
**Dominant hand**		0.488	0.8
Right	310 (86.6)	251 (86.9)	
Left/AMB	48 (13.4)	38 (13.1)	
**Orientation**			0.112
Posterior	306 (85.5)	256 (88.6)	
Anterior	52 (14.5)	33 (11.4)	

The correlation matrix revealed minor relationships between pain intensity and key demographic variables. Pain intensity was negatively correlated with age (r = -0.07) and orientation (r = -0.12), while it showed a slight positive correlation with sex (r = 0.09), suggesting that girls might experience slightly higher pain intensity than boys. Dominant hand showed a negligible negative correlation with pain intensity (r = -0.04), as seen in Table 3.

**Table 3:** Correlation matrix: pain intensity vs. key variables

Variable	Pain intensity	Age	Sex	Dominant hand	Orientation
Pain intensity	1	–0.07	0.09	–0.04	–0.12
Age	–0.07	1	–0.02	0.01	–0.06
Sex (M = 1, F = 2)	0.09	–0.02	1	–0.02	0.03
Dominant hand (R = 1…)	–0.04	0.01	–0.02	1	–0.09
Orientation (Ant = 1, P = 2)	–0.12	–0.06	0.03	–0.09	1

Logistic regression analysis was conducted to assess the likelihood of experiencing pain based on various predictors. The model included age, sex, orientation, and dominant hand as predictors but found none to be significantly associated with the presence of pain. The odds ratios were close to 1 for all predictors: age (OR = 0.97; 95% CI, 0.90–1.05), sex (male vs. female, OR = 1.14; 95% CI, 0.83–1.58), posterior orientation (OR = 1.21; 95% CI, 0.79–1.86), and dominant hand (right, OR = 0.98; 95% CI, 0.58–1.64), with *P* values all above 0.35. The chi-squared value of the model was 3.74 with a *P* value of 0.442, indicating a poor fit, and a Nagelkerke R^2^ value of 0.012, which further confirmed the minimal explanatory power of these variables for predicting pain presence (Table 4).

**Table 4:** Logistic regression for the presence of pain

Predictor	OR	95% CI	*P* value
Age (years)	0.97	0.90 – 1.05	0.428
Sex (Male vs. Female)	1.14	0.83 – 1.58	0.403
Posterior orientation	1.21	0.79 – 1.86	0.356
Dominant hand (Right)	0.98	0.58 – 1.64	0.931

Dependent variable: Pain (1 = yes, 0 = no); Model χ^2^ = 3.74 (df = 4), P = 0.442; Nagelkerke R^2^ = 0.012.

We monitored the postural evolution of the subjects by applying plantar corrections to a cohort of 647 individuals. Among these, 178 patients returned for pain reassessment, of whom 137 underwent re-evaluation on a stabilometric platform. A predominantly posterior pressure distribution was observed in 86.86% of the initial group of 647 patients. Consequently, eight patients with anterior orientations were excluded, leaving 129 subjects with posterior orientations for analysis. The pain distribution among the returning 178 patients was as follows: 47% reported no pain, 32% experienced moderate pain, 16% mild pain, and 5% intense pain. Those in the no-pain group returned primarily due to interest in preventative check-ups. All 178 patients consented to undergo treatment via plantar correction.

**Figure 1:**
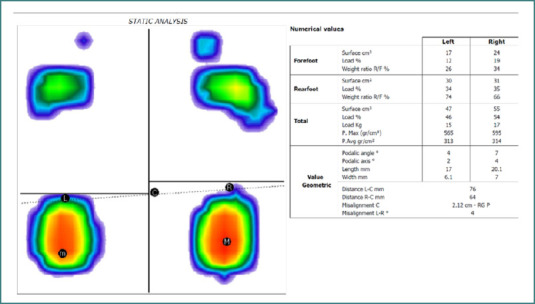
Impact of personalised plantar insoles on alleviating growing pain

**Figure 2:**
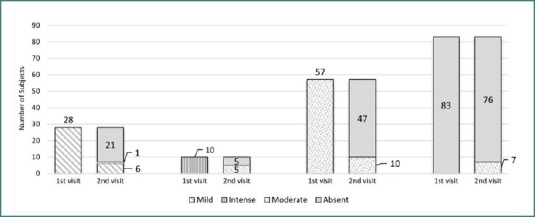
Impact of personalised plantar insoles on alleviating growing pain

The impact of personalized plantar insoles on alleviating growing pains is depicted in Figures 1 and 2. These figures illustrate the experimental and statistical validation of the hypotheses underpinning this research. For example, among the 28 patients experiencing mild pain at follow-up, 21 reported no pain, one exhibited moderate pain, and six continued to experience mild pain. Of the ten patients initially experiencing intense pain, half reported no pain following treatment, while the remaining half transitioned to moderate pain. A notable improvement was observed in the group initially presenting with moderate pain, with 47 patients reporting no pain at follow-up and 10 continuing to experience moderate pain. Of the 83 patients who initially reported no pain, seven developed moderate pain during the follow-up period, indicating varied individual responses to the intervention.

Outcomes of plantar correction were assessed in 129 children with a posterior orientation. Among the 70 children who had pain at baseline, 41 (59%) showed improvement, 10 (14%) remained stationary, and 19 (27%) worsened. For the 59 children without baseline pain, improvement was seen in 31 (53%), stationary status in 14 (24%), and worsening in 14 (23%), as seen in Table 5.

**Table 5:** Plantar correction outcomes in reassessed posterior-orientation children (*n* = 129)

Baseline pain status	Improved FPD	Stationary	Worsened FPD	Total
**Pain present (*n* = 70)**	41 (59%)	10 (14%)	19 (27%)	70
**No pain (*n* = 59)**	31 (53%)	14 (24%)	14 (23%)	59

**Figure 3:**
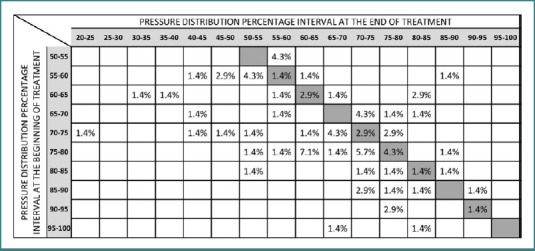
Effects of plantar correction on FPD patients with pain

Figure 3 documents the effects of plantar correction on FPD among patients who initially reported pain (*n* = 70). It was found that 59% of these patients experienced improvement, as indicated by their positioning on the left side of the grey diagonal boxes. Within the stationary zone, 14% of the participants demonstrated no change in FPD values, maintaining consistent measurements across assessments. Conversely, the right side of the grey diagonal boxes, defined as the aggravation zone, included 27% of the subjects who exhibited worsened conditions post-intervention.

**Figure 4:**
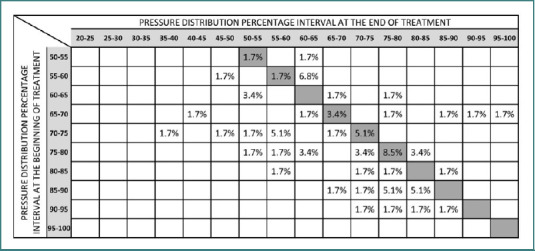
Effects of plantar correction on FPD patients without pain

Figure 4 presents the impact of plantar correction on FPD among a control group of pain-free patients (*n* = 83) who underwent reassessment; of these, 59 completed a repeat assessment on the platform. An improvement was noted in 53% of the patients, as reflected by their data on the left side of the table. Meanwhile, 24% of the participants remained in the stationary zone, retaining unchanged FPD values. Additionally, 23% of the subjects were categorized within the aggravation zone, indicating a deterioration in their foot pressure distribution following plantar correction. This comparative analysis highlights the varied responses to plantar correction observed between patients with and without initial pain.

Among 178 reassessed children, 62 were categorized as 'Posterior + High' compliance (G1), 54 as 'Posterior + Low' (G2), 32 as 'Anterior + High' (G3), and 30 as 'Anterior + Low' (G4) (Table 6). Mean ages ranged from 7.6 ± 2.0 years (G4) to 8.5 ± 2.5 years (G2), with no statistically significant differences (*P* = 0.394). In G1, 44 children had baseline pain, and 36 of these (81.8%) reported pain improvement at follow-up; in G2, 40 had baseline pain, and 25 (62.5%) improved; in G3, 22 had baseline pain, and 16 (72.7%) improved; in G4, 24 had baseline pain, and 13 (54.2%) improved (*P* = 0.021). Regarding foot-pressure distribution (FPD), 42 of 62 children in G1 (67.7%) showed objective improvement in platform measurements, compared to 30 of 54 (55.6%) in G2, 18 of 32 (56.3%) in G3, and 14 of 30 (46.7%) in G4 (*P* = 0.043). Sex distribution (46.7–53.2% males) and baseline pain severity categories (no pain, mild, moderate, intense) appeared similar across groups, with *P* values above 0.05.

**Table 6:** Subgroup analysis of orientation and insole compliance in reassessed children (*n* = 178)

Variable	G1 Posterior + High (*n* = 62)	G2 Posterior + Low (*n* = 54)	G3 Anterior + High (*n* = 32)	G4 Anterior + Low (*n* = 30)	*P* value
**Age (years, mean ± SD)**	8.1 ± 2.2	8.5 ± 2.5	7.9 ± 2.1	7.6 ± 2.0	0.394
**Sex (Males, n (%))**	33 (53.2)	28 (51.9)	15 (46.9)	14 (46.7)	0.781
**Baseline pain status**					
– No pain (*n* (%))	18 (29.0)	14 (25.9)	10 (31.3)	6 (20.0)	0.662
– Mild pain (*n* (%))	16 (25.8)	12 (22.2)	9 (28.1)	10 (33.3)	0.824
– Moderate pain (*n* (%))	22 (35.5)	20 (37.0)	8 (25.0)	11 (36.7)	0.531
– Intense pain (*n* (%))	6 (9.7)	8 (14.8)	5 (15.6)	3 (10.0)	0.374
**Pain improvement † (*n* (%))**	36 (81.8)	25 (62.5)	16 (72.7)	13 (54.2)	0.021*
**FPD improvement (*n* (%))**	42 (67.7)	30 (55.6)	18 (56.3)	14 (46.7)	0.043*

† Pain improvement rates calculated among those with baseline pain only. *P < 0.05 indicates statistical significance; Posterior vs. Anterior: Based on whether hindfoot or forefoot pressures dominated at baseline. High vs. Low Compliance: Self-reported daily insole wear above or below a designated cutoff (e.g., ≥8 vs. <8 hours/day). Pain improvement: Percentage of children who reported reduced pain severity at follow-up among those with mild, moderate, or intense baseline pain. FPD improvement: Percentage of children demonstrating improved foot-pressure distribution on follow-up stabilometric testing (e.g., moving closer to a balanced load).

A total of 83 children initially reported no pain, and 95 reported pain at baseline. They underwent gait analysis at two time points (baseline and 2–6 months later) (Table 7). In the no-pain group, step length increased from 50.2 ± 5.6 cm to 53.1 ± 5.7 cm (*P* = 0.023), and gait velocity rose from 1.06 ± 0.10 m/s to 1.15 ± 0.12 m/s (*P* = 0.012). Stance width (14.6 ± 2.3 cm to 15.0 ± 2.2 cm, *P* = 0.281), foot progression angle (6.3 ± 3.0° to 5.8 ± 2.8°, *P* = 0.367), and single-limb stance (0.44 ± 0.05 s to 0.46 ± 0.06 s, *P* = 0.144) showed no statistically significant changes. Among those with baseline pain, step length increased from 49.0 ± 6.2 cm to 52.5 ± 6.0 cm (*P* = 0.016), stance width from 14.9 ± 2.4 cm to 15.6 ± 2.3 cm (*P* = 0.048), single-limb stance from 0.42 ± 0.06 s to 0.45 ± 0.07 s (*P* = 0.041), and gait velocity from 0.97 ± 0.11 m/s to 1.09 ± 0.15 m/s (*P* < 0.001). The foot progression angle decreased from 6.7 ± 3.2° to 5.9 ± 2.7° (*P* = 0.082) but did not reach statistical significance.

**Table 7:** Gait parameter changes in reassessed children (*n* = 178) by pain status

Gait parameter	No pain baseline (*n* = 83)	No pain follow-up	*P* value	Pain baseline (*n* = 95)	Pain follow-up	*P* value
**Step length (cm)**	50.2 ± 5.6	53.1 ± 5.7	0.023 **	49.0 ± 6.2	52.5 ± 6.0	0.016 **
**Stance width (cm)**	14.6 ± 2.3	15.0 ± 2.2	0.281	14.9 ± 2.4	15.6 ± 2.3	0.048 **
**Foot progression angle (°)**	6.3 ± 3.0	5.8 ± 2.8	0.367	6.7 ± 3.2	5.9 ± 2.7	0.082
**Single-limb stance (s)**	0.44 ± 0.05	0.46 ± 0.06	0.144	0.42 ± 0.06	0.45 ± 0.07	0.041 **
**Gait velocity (m/s)**	1.06 ± 0.10	1.15 ± 0.12	0.012 **	0.97 ± 0.11	1.09 ± 0.15	<0.001 **

No pain baseline: Children who initially reported no leg pain. Pain baseline: Children who initially reported mild, moderate, or intense leg pain. Follow-up: Conducted approximately 2–6 months after baseline, coinciding with stabilometric reassessment. All values are presented as mean ± SD. P values reflect within-group comparisons (baseline vs. follow-up). Gait parameters were measured via a computerized gait-analysis system. P values (<0.05) indicate statistical significance.

## DISCUSSION

### Interpretation of findings

This investigation sought to evaluate the impact of custom insoles on pain reduction and foot pressure distribution (FPD). Utilization of personalized insoles may facilitate postural adjustments in static and dynamic stances due to varying adaptation strategies [1,30]. Notably, a significant observation from this study was the increase in rearfoot pressures in 86% of patients following insole treatment, an outcome that underscores the potential of insoles in modifying plantar load distribution. The employment of a stabilometric platform was justified by its demonstrated intra- and inter-session reliabilities [31], enhancing the credibility of posturostabilometric assessments. This platform proved instrumental not only in reliably assessing posture but also in providing insights into postural dynamics among children with pain or atypical body positions during static and dynamic activities. The findings advocate for more comprehensive studies employing stringent research methodologies to solidify these preliminary observations and to further elucidate the link between stabilometric assessments and postural adjustments in children experiencing growth-related pain.

The therapeutic efficacy of insoles was evidenced by the improvement or resolution of pain, corroborating previous research that supports the role of postural insoles in diminishing pain severity and its interference with daily activities [32]. Custom plantar corrections were observed to ameliorate posterior FPD in the participants, which aligns with the anticipated outcomes of mitigating abnormal lower-extremity alignment and the consequent stress on the musculoskeletal system. Such stress is implicated in several orthopedic conditions, including plantar fasciitis and knee disorders [20]. The average age of participants was 8.14 years, with pain observed across all age categories, consistent with existing literature on the prevalence of growing pains within the 4–14 year age spectrum [18,25]. Despite this, the study did not reveal significant correlations between the intensity of pain, FPD, and demographic factors such as sex and age, which aligns with findings from other research [3,20,25,33]. These insights suggest that while plantar pressure distribution does not significantly correlate with age, the symmetry in the distribution of pain and FPD across dominant hands remained consistent among subjects.

Patient compliance and satisfaction with foot orthoses have generally been high, particularly in managing painful foot conditions [34]. In this study, children with postural discrepancies who received appropriate plantar corrections during stabilization exhibited significant reductions or complete alleviation of growth-related pain. The pain intensity, categorized subjectively, either decreased or stabilized, which supports the non-detrimental effect of the insoles on the majority of the participants. Nevertheless, the minor group reporting persistent mild pain might indicate potential non-adherence to the insole usage guidelines, suggesting a variable that could affect other participants as well.

Moreover, despite high FPD percentages reported in both pain and no-pain groups, these figures likely reflect more than mere non-compliance; they may also be influenced by the body's adaptation to additional sensory inputs [35,36]. This study acknowledged the possible interactions between posture and other physiological systems, such as the stomatognathic [36,37] and visual systems [37], which could also affect postural control. These findings highlight the complex interplay of biomechanical and sensory factors in managing pediatric foot and posture-related conditions.

### Study limitations and future perspectives

Despite these promising observations, our results must be interpreted in the context of certain limitations. First, as a retrospective observational study, we did not randomize participants or control for extraneous variables. Second, self-reported adherence to insoles was not systematically monitored, which could influence FPD changes. Third, the natural course of “growing pains” sometimes resolves spontaneously, confounding the true effect of posture correction. Nevertheless, our data indicate that posterior orientation is common in children with or without reported leg pain, and insoles can affect many positive changes in foot pressure distribution. Future prospective controlled trials are warranted to test whether targeted anteroposterior corrections can consistently alleviate growing pains and reduce the incidence of malalignment-related complaints. These efforts should include objective compliance tracking and standardizing re-evaluation intervals to refine postural intervention protocols.

## CONCLUSION

This study underscores the feasibility and potential benefit of individualized plantar correction in children exhibiting posterior foot loading and growing pains. Despite the absence of strong demographic or orientation-based predictors of pain, over half of the re-evaluated participants displayed measurable improvement in foot pressure distribution. A concomitant decrease in subjective pain levels was also noted in many. These results highlight the need to investigate the nuanced interplay between foot posture and pediatric musculoskeletal discomfort. Proper plantar corrections appear to be a worthwhile adjunct to conventional management of growing pains. By recognizing that not all children respond similarly to orthotic therapy, and some may even show inconsistent or worsened FPD, clinicians can refine follow-up strategies and consider additional sensory or postural inputs (occlusion, vision, or vestibular function) that shape the child’s dynamic posture. Future controlled studies are necessary to delineate how these interventions truly contribute to resolving growing pains and improving long-term musculoskeletal health.

## Personal thanks

Publication of this paper was supported by Carol Davila University of Medicine and Pharmacy, through the institutional program “Publish not Perish”.
